# Ameloblastic fibro-dentinoma: a rare mixed odontogenic tumor case report with review of literature

**DOI:** 10.1186/s43046-023-00193-0

**Published:** 2023-10-30

**Authors:** Nihal Mohamed Ahmed Darwish, Hatem Wael Abdel-Fattah Amer, Nesma Nabil Mohamed Mahrous

**Affiliations:** Oral and Maxillofacial Pathology, Faculty of Dentistry, Cairo University, Cairo, Egypt

**Keywords:** Ameloblastic fibro-dentinoma, Ameloblastic fibroma, Ameloblastic fibro-odontome, Mixed odontogenic tumors, Case report

## Abstract

**Background:**

Ameloblastic fibro-dentinoma is considered a rare, benign, mixed odontogenic tumor that occurs mainly in the posterior mandible in the 1st–2nd decade of life. Although the clinical behavior of Ameloblastic fibro-dentinoma is similar to that of ameloblastic fibroma, there is a debate about whether Ameloblastic fibro-dentinoma is a developing hamartomatous odontoma or a separate neoplastic odontogenic tumor like ameloblastic fibroma. However, it is important to understand the histopathogenesis of this rare tumor.

**Case presentation:**

A case report presenting an 11-year-old male child with a swelling in the posterior mandible. Radiographic examination revealed a multilocular lesion with mixed radiodensity related to the impacted lower left second premolar tooth. Incisional biopsy was done, and microscopic examination revealed cords and nests of odontogenic follicles lined by ameloblast-like cells and central stellate reticulum-like cells in the primitive ecto-mesenchymal stroma with areas of dentinoid material and osteodentin. The diagnosis was ameloblastic fibro-dentinoma. Surgical excision of the lesion was done, and the patient was followed up for 1 year without evidence of recurrence.

**Conclusion:**

Reporting such a rare entity clarifies the debate about its nature and the importance of early diagnosis of lesions that are associated with unerupted teeth showing how it is effective in early management and prognosis.

## Background

Ameloblastic fibro-dentinoma (AFD) accounts for 2% of all odontogenic tumors. It occurs most frequently at a young age, with the mean patient age being 15 years. This tumor predominantly affects the mandible especially the posterior region more than the maxilla with slight male predilection [1, 2] (WHO, 2022). AFD is very similar to ameloblastic fibroma (AF) and ameloblastic fibro-odontoma (AFO). Clinically, it is a slow-growing asymptomatic tumor, it may be associated with impacted or unerupted teeth additionally it could cause cortical perforation. Radiologically, it appears as a well-defined radiolucency with varying degrees of radiopacity [3].

Histologically AFD is composed of odontogenic epithelium, immature connective tissue, and is characterized by the formation of dysplastic dentin [3]. The odontogenic epithelium is in the form of strands and small islands like dental lamina and enamel organs in a highly cellular primitive ectomesenchyme like dental papillae. Tall cuboidal to columnar cells with central stellate reticulum-like cells similar to ameloblastic follicles may be seen lined with a few odontogenic strands or follicles. Osteodentin, dentinoid, and tubular dentin deposition may be evident in relation to the odontogenic epithelium [4]. Enamel or enamel matrix must not seen in cases of AFD [3].

There is a debate about AFO and AFD as they appear to be intermediate between AF and odontoma. The WHO classification in 2005 classified the AFDs and AFOs under AFs however, further studies disapprove of this and concluded that AFs should be regarded as a true neoplasm whereas AFO is more likely a developing odontoma[3, 5–7]. The continuum concept with AF, AFO, and odontomas was rejected on the basis of age of occurrence, site of occurrence, histopathology, gender, and evidence studied in recurrent cases [8].

In the WHO 2017 edition and current WHO tumors classification 2022, AFD and AFO are classified as developing odontomas (hamartomatous process). Although the presence of BRAF and V600E mutations in AFD and AFO is similar to AF but absent in odontoma, supported the arguments that at least some of these lesions are in fact neoplastic, particularly those with locally aggressive biological behavior, large size, and recurrence [8–11]

### Case presentation

**Fig. 1:**
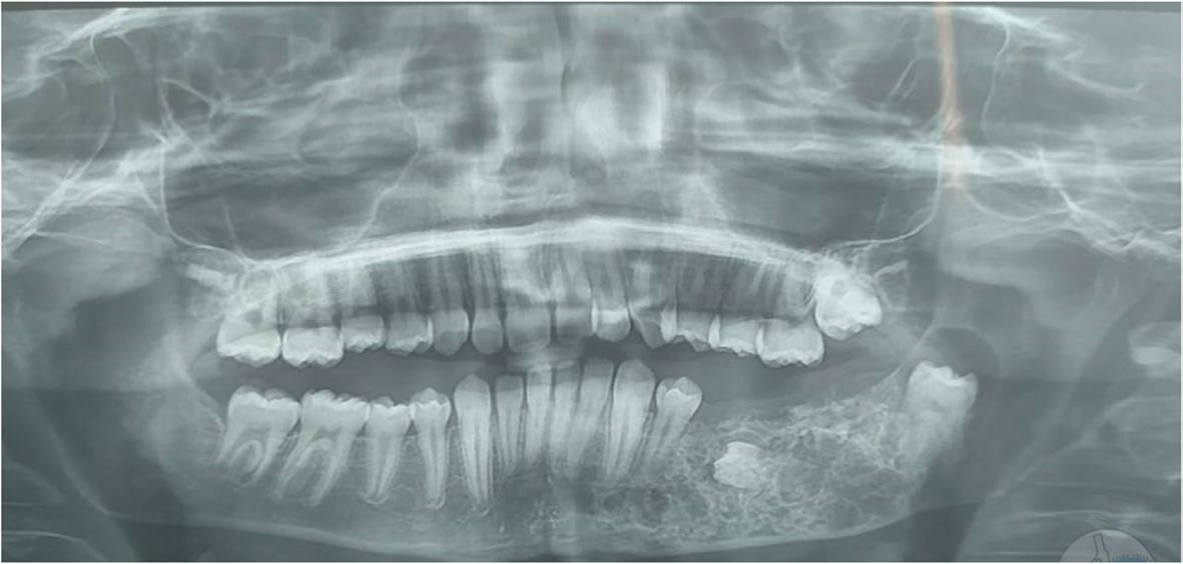
Preoperative panoramic radiograph revealed a well-defined osteolytic multilocular radiolucency at posterior mandible associated with diffuse radiopacities causing displacement of teeth and hindering their eruption

An 11-year-old male child presented to the oral and maxillofacial surgery department, faculty of dentistry, Cairo University with a swelling in the posterior mandible. Radiographic examination revealed a multilocular lesion with mixed radiodensity related to the impacted lower left second premolar tooth. The lesion was large in size extending from the canine to the molars area causing teeth displacement and hindering the eruption of the lower 2nd premolar and one of the lower molars (Fig. 1).

Based on the clinical and radiographic findings, differential diagnoses of odontogenic tumors: ameloblastoma, ameloblastic fibroma, ameloblastic fibro-odontoma, or ameloblastic fibro-dentinoma were made.

**Fig. 2:**
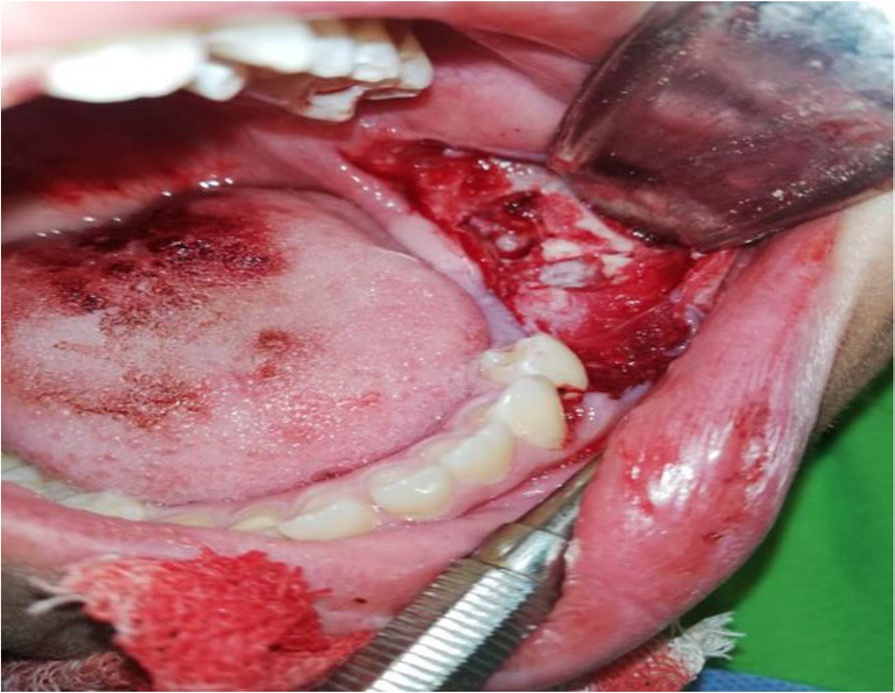
An intraoperative clinical photo showing the incision through the intraoral bony swelling

**Fig. 3:**
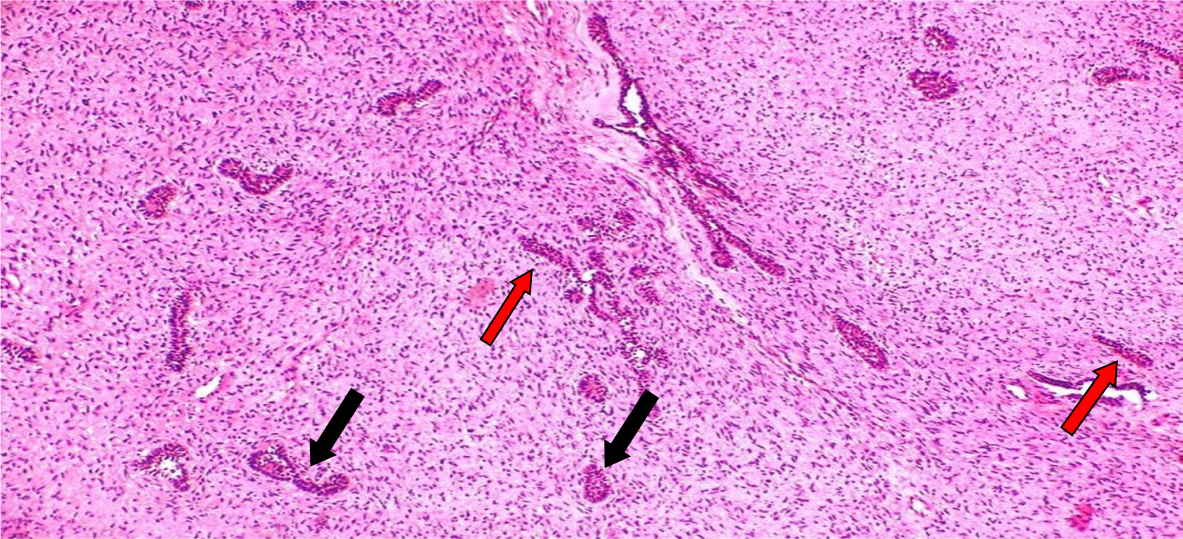
A photomicrograph of Hematoxylin and eosin (H&E) stained sections showing cords (red arrows) and nests (black arrows) of odontogenic epithelium in highly proliferative primitive ectomesenchymal stroma (× 100)

**Fig. 4:**
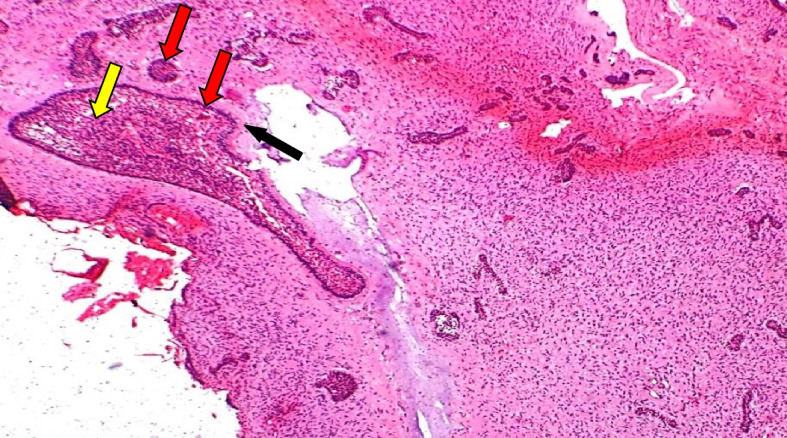
A photomicrograph of Hematoxylin and eosin (H&E) stained sections showing the cords and nests of odontogenic follicles lined by ameloblast-like cells (red arrows) and central stellate reticulum-like cells (yellow arrows) and surrounded by juxta epithelial hyalinization (black arrow). It also shows highly proliferative ectomesenchymal primitive cells in the surrounding stroma

**Fig. 5:**
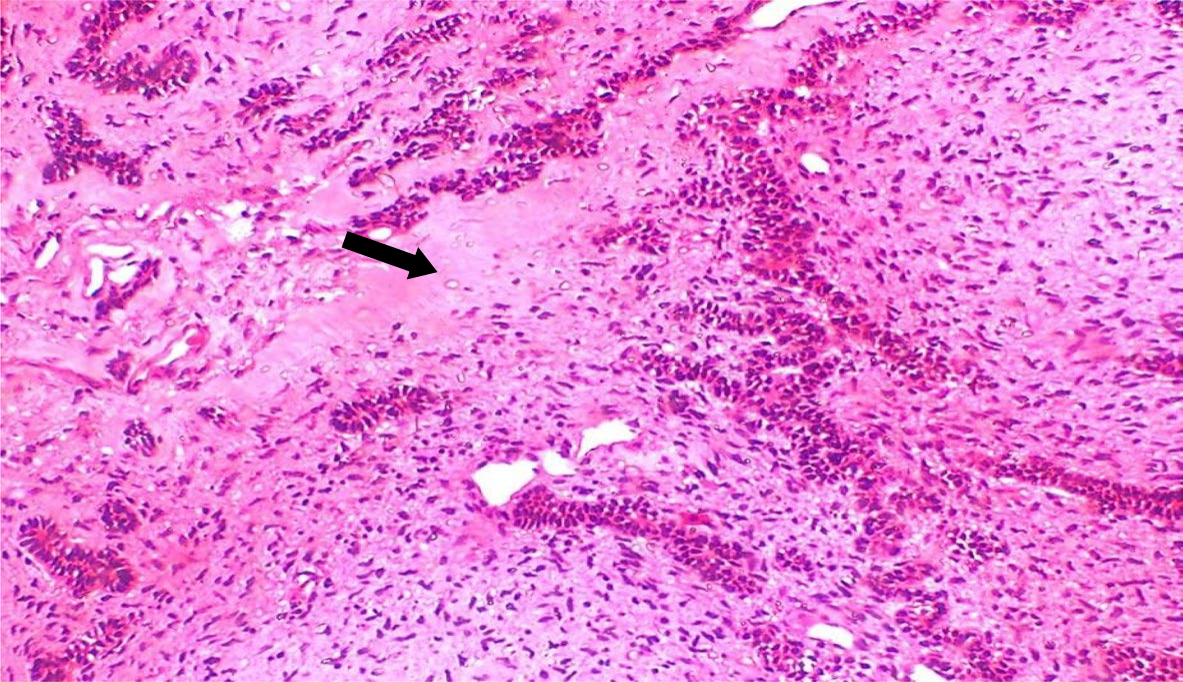
A photomicrograph of Hematoxylin and eosin (H&E) stained sections showing cords of odontogenic epithelium. The surrounding connective tissue stroma showed primitive ectomesenchymal cells with areas of dentinoid material (black arrow) (× 200)

**Fig. 6:**
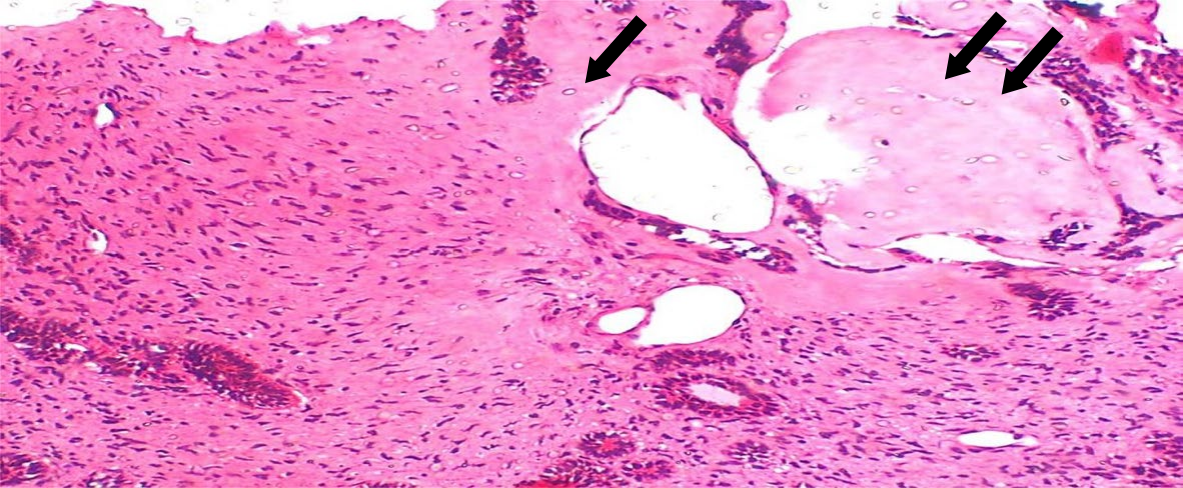
A photomicrograph of Hematoxylin and eosin (H&E) stained sections showing the surrounding connective tissue primitive stroma with areas osteodentin (black arrows) (× 400)

**Fig. 7:**
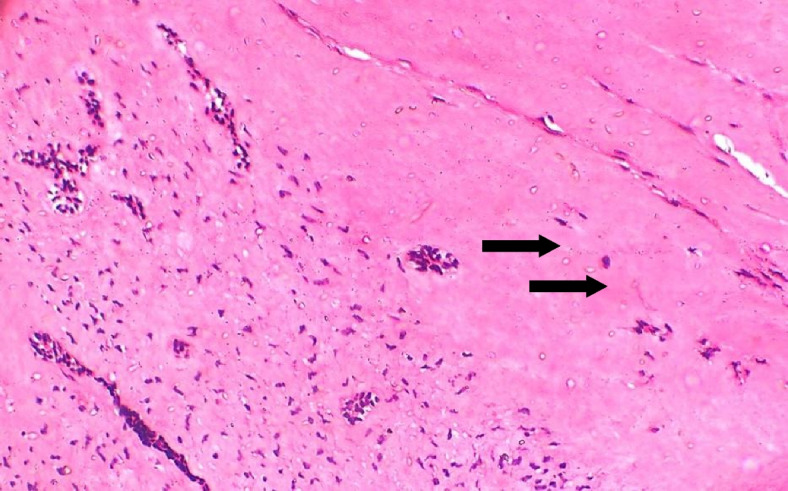
A photomicrograph of Hematoxylin and eosin (H&E) stained sections showing large areas of dysplastic dentin (× 400)

Incisional biopsy was done (Fig. 2) and referred to the oral and maxillofacial pathology department, faculty of dentistry, Cairo University, and the microscopic examination revealed cords and nests of odontogenic follicles like enamel organs lined by tall cuboidal to columnar cells with central stellate reticulum-like cells similar to ameloblastic follicles surrounded by juxta epithelial hyalinization (Figs. 3 and 4). The surrounding connective tissue stroma showed primitive immature ectomesenchymal cells resembling dental lamina with areas of dentinoid material (Fig. 5 black arrow) and osteodentin (Figs. 6 and 7 black arrows). The microscopic features were consistent with ameloblastic fibro-dentinoma. Surgical excision of the lesion was done, and the patient was followed up for 1 year without evidence of recurrence.

## Discussion

AFs, AFDs, AFOs, and odontomas are lesions that almost share the same histopathological, clinical, and radiographical features this in turn results in an argument about whether they can be categorized as a separate pathological entity or as developmental stages of the same lesion. That is why some researchers consider AFs and odontomas to be the extremes while, AFDs and AFOs are intermediate stages in between them [7, 8]. Philipsen et al. supposed that AF and AFD are expressed in two forms. The neoplastic one if left more time without treatment will not mature into odontoma. The other variant is a hamartomatous lesion that has the potential to mature into an AFO and complex odontoma. Gardner has an opinion that the AFD should be referred to simply as AF [12].

In 2005, WHO classified the AFDs under AFs. The recent WHO classification (2017) and (2022) considered AFDs to be a hamartomatous process supposing that once the hard tissue is formed, they mature to form odontomas [10, 11]. However, cases have been reported with significant large growth patterns causing cortical plate perforation. Moreover, many reports of cases with malignant transformation and recurrence have been reported. Additionally, AF, AFO, and AFD harbor the same BRAF p.V600E mutation in 46%, 34%, and 69% of cases respectively [8, 13, 14].

AFD is considered is a rare mixed odontogenic tumor that arises mainly in the posterior region mandible and is usually with an unerupted molar which in accordance with the present case too. AFD is more commonly reported in males as in this case. AFD often grows with no symptoms and may reach large sizes [13].

Histologically, the tumor is characterized by epithelial and ectomesenchymal neoplastic components. The epithelial component consists of odontogenic nest or cords lined by tall ameloblastic-like cells and central stellate reticulum-like cells. The ectomesenchymal component resembles the dental lamina with dysplastic dentin deposition.

AFD could be distinguished from other similar lesion like AF and AFO by that AFD exhibit dysplastic or tubular dentin, whereas enamel matrix deposits are found in the AFO but, regarding AF there is no any type of dental hard tissue deposits [8]. In this case, microscopically, we observed long narrow cords and islands of odontogenic follicles with juxtaepithelial hyalinization. The epithelial strands resided in a primitive ectomesenchymal stroma with stellate-shaped fibroblasts exhibiting long slender cytoplasmic extensions that resembled dental papilla. The lesions exhibited non-tubular dentin entrapping cells in multiple lacunae in close relation to odontogenic epithelium resembling dentinoid. The presence of dentinoid excludes AF tumor. Additionally, there is no enamel matrix or enamel spaces, so AFO was excluded. All of these features are consistence with the AFD.

As reported by Gardner and Farquhar, in AFDs different forms of dentin could be seen such as osteodentin, dentinoid, and dentin undergoing globular mineralization [15]. All of this tissue is considered dentinal induction; however, the juxtaepithelial hyalinization is categorized as non-dentinal induction in the ecto-mesenchyme [16]. In the present case, these features are present in the form of dentenoid material, osteodentin, and juxtaepithelial hyalinization surrounding the odontogenic follicles and cords.

Since AFDs have with low recurrence rate and usually with good biological behavior a conservative approach is usually recommended for this tumor [17]. In 2012, Giraddi, G.B., and V. Garg reported an aggressive atypical AFD presented with resorption and perforation of the cortical plate which was treated radically treated [4]

Occasionally, ameloblastic fibro-dentinosarcoma arises from the malignant transformation of AFD [18]. The 2022 WHO classification of odontogenic sarcomas presented three tumors: ameloblastic fibro-sarcoma, ameloblastic fibro-dentinosarcoma, and ameloblastic fibro-odontosarcoma. They arise from the malignant transformation of the mesenchymal component while the epithelial component does not show any malignant changes. In the present case the lesion was large in size with no evidence of malignant transformation so, conservative surgical treatment was done with a follow-up of 1 year.

## Conclusion

This paper is reporting on one of the rare entities with illustrating the historical and current debate about it. Additionally, the presented case showed the importance of early diagnosis of lesions related to the delayed eruption or missing teeth and how it’s effective in early management and hence prognosis of the patient.

## Data Availability

The data used during the current study are available from the corresponding author on reasonable request.

## Abbreviations

AFD: Ameloblastic fibro-dentinoma
AF: Ameloblastic fibroma
AFO: Ameloblastic fibro-odontoma
WHO: World Health Organization
